# The Motivation MAP: an exercise-message framework to foster positive affect, challenge all-or-nothing thinking, and prioritize self-care

**DOI:** 10.3389/fpsyg.2024.1441844

**Published:** 2024-10-22

**Authors:** Michelle L. Segar

**Affiliations:** The Institute for Research on Women and Gender, University of Michigan, Ann Arbor, MI, United States

**Keywords:** motivation, exercise, physical activity, affect, motivation states, framing, messages, dual-process

## Introduction

Why do so many people, despite knowing the potent benefits from exercise and having the best of intentions, quickly lose their motivation? Curiosity about this phenomenon ignited my career in the science and practice of cultivating sustainable exercise motivation and participation, and has sustained it for 30 years.

In 1994, I was confronted with an unanticipated problem while conducting my Kinesiology master's thesis (Segar et al., 1998): The cancer survivors who had been motivated to sustain exercise during the study *for our research* lost their motivation to stick with it *just for themselves* when the study ended.

This conundrum showed how deeply this problem was embedded within our cultural psyche. I needed to learn more: getting an MPH in health-behavior intervention design and evaluation, testing out and refining my ideas through real-world delivery of a theory-based intervention, and eventually getting a doctorate in personality psychology, the original home of motivation scholarship.

This psychological perspective brought me back to my original epiphany: the need for a map that enables people to free themselves from the cultural expectations that lead to repeated motivation failures. I now call this The Motivation MAP, a flexible motivation framework that utilizes three strategic messages to reframe exercise and cultivate positive new exercise beliefs, meanings, and experiences to support increased, sustainable movement among inactive individuals and populations.

Strategic messaging is a framing tactic to shape perceptions and persuade behavior (The Framing Institute, 2005). Frames are rooted in culture and influence how people see a behavior, including their beliefs about why and how to do it (Cooke-Jackson and Rubinsky, 2023; Gray and Harrington, 2011; Smith et al., 2010). Our frames influence what a behavior means to us and how we experience doing it (e.g., Does exercise feel hard or easy? Is exercise a chore or a gift?), thus exercise frames constitute a powerful cognitive map with affective and motivation consequences (Segar et al., 2016).

While research exists about exercise messaging strategies, similar to exercise science more generally, it has been dominated and limited by theoretical frameworks emphasizing rationality and conscious reflection (Brand and Ekkekakis, 2017). With the recent emergence of science on motivation states and dual-process theories' emphasis on exercise affective experiences (Kwan et al., 2017; Stults-Kolehmainen et al., 2023) there is a critical need for messaging and framing research to incorporate this new science.

I constructed The Motivation MAP to help inactive people reframe the meaning of exercise, reorienting it in ways that theory, science, and experience show cultivate positive affective experiences exercising and support the ability to fit in, prioritize, and regulate physical activity. The pilot version of The Motivation MAP was developed in 1997 for real-world commercial use. It has continued to be refined with scientific advancements and ongoing implementation, and others have recently started using it in federally funded behavioral interventions. Emerging theory, mounting science, and decades of real-world experience offer support for the unique contribution that The Motivation MAP can make to next-generation exercise research, hypothesis testing, and behavioral outcomes.

## The Motivation MAP

When people are psychologically oriented toward exercise in maladaptive ways, it is critical to reorient them to more adaptive ways of thinking.

The acronym MAP reflects the framework's three-part reframing structure: Meaning, Awareness, and Permission to Prioritize. It can be difficult for people to truly understand the psycho-social exercise context that they live within. Thus, The Motivation MAP and its underlying methods guide people to critically evaluate their past exercise beliefs and experiences and invite them to cognitively and experientially reorient toward exercise.

The Motivation MAP's messages ask people to reflect on what they've learned from society about *why* and *how* they should exercise and gives them permission to reframe exercise, opening a cognitive door for rethinking their exercise motives, approaches to exercising, and priorities. This shift in thinking is concurrently accompanied by methods that invite people to experiment with participating in new physical activities, intensities, durations, and contexts in order to cultivate new positive affective experiences during exercise. Specifically, it aims to influence three hypothesized mediators of change: 1. Exercising in pleasurable ways; 2. Believing all movement is valid and worth doing; and 3. Perceiving exercise as self-care and prioritizing time for self-care and exercise. As a whole, The Motivation MAP intervention constitutes an embodied resocialization process, empowering people to transform their mindset about and relationship with exercising and movement (Segar, 2015; Segar et al., 2002).

### Meaning

Much of what we experience has a deep, complex personal meaning for us that influences our behavioral choices across life arenas (Chan et al., 2021; Spruijt-Metz, 1995).

For many, especially inactive individuals, the objective meaning of exercising to improve health is often complicated by below-the-surface meaning of exercise as a painful, inconvenient, body-shaping, and obligatory “chore” (Segar et al., 2017a, 2007). As a result, despite an initial deep commitment, many quickly lose their motivation and stop exercising.

***Message #1***: “***Move in ways that feel good to you***.”

The *Move in ways that feel good to you* message gives people permission to move away from exercise plans that feel self-defeating and instead choose physical activities that are personally inspiring, intrinsically motivating, and positively self-affirming. This message strategy encourages people to reframe physical movement as a daily option for cultivating positive affective experiences that they like and want (i.e., converting it into a “gift” they want to give themselves; Berridge et al., 2009).

#### Empirical support

Decision-making science suggests that it is how people expect to *immediately* feel from their behavioral choices, not the *future* value of these choices, that better drives their in-the-moment decisions (Chang and Tuan Pham, 2013). Exciting new science and dual-process models of physical activity (Brand and Ekkekakis, 2017; Conroy and Berry, 2017; Kwan et al., 2017) support the high value of helping people learn to have and expect positive experiences exercising, such as *feeling good*, generally proposing that positive affective experiences during physical activity result in a “greater likelihood of future physical activity behavior” (Stevens et al., 2020).

### Awareness

Giving oneself *permission to exercise in ways that feel good* requires Awareness of our social conditioning regarding what “counts” as exercise. Until recently, “threshold messages” (communications prescribing specific amounts, durations, and intensities) have dominated the exercise messaging space in everything from PSAs to clinician advice (Segar et al., 2020). Yet, threshold messaging is not evidence-based (Warburton and Bredin, 2016) and may unintentionally create all-or-nothing standards for exercising that thwart decisions to move (Arena et al., 2018; Knox et al., 2014). When people believe they have to do it “right” to count and “right” isn't feasible, they often believe it's not worth doing at all (Segar, 2022). Because threshold messages emphasize *achieving* criteria, they may also inadvertently prevent people from seeking feel-good *experiences* through exercise.

To internalize the first “*feel-good*” Motivation MAP message, people need to gain Awareness about the newest physical activity recommendations suggesting that when it comes to movement, everything counts and adds up (Bull et al., 2020).

***Message #2***: “***Everything counts. It all adds up***.”

The “Everything counts. It all adds up” message guides people to start believing that all activity—every step, every minute of moving—*counts* as exercise, adds up, and is worthwhile. This message signals that no single movement, including housecleaning, shopping, or standing up should be discounted nor devalued (Arena et al., 2018).

This message reduces real-life constraints, increases competence, and validates claiming micro-movements, integrating with the *Move in ways that feel good to you* message to help people transform their overarching Meaning for exercise from a “*chore* to a *gift”* (Segar, 2015).

#### Empirical support

Accumulating evidence suggests that messages teaching more inclusive and flexible definitions of movement are associated with better outcomes than traditional threshold messages (Mailey and Hsu, 2019; Walters et al., 2022; Zahrt and Crum, 2020). The *Everything Counts* messaging strategy is supported by the physical activity recommendations' removal of the 10-minute bout criterion, which aligned our global promotion of physical activity with behavioral science, helping people more easily integrate movement into their daily life (Arena et al., 2018).

Leading-edge research finds that motivation states are dynamic, random, and transient throughout the day, and influence self-regulation (Stults-Kolehmainen et al., 2023). Thus, cultivating *Everything Counts* thinking may help people better notice, and act on, numerous daily internal and external cues that encourage movement but which they had not previously felt nor sufficiently valued. Furthermore, if people *feel positive* from or about their choice to claim these micro-movement opportunities, this positivity may further increase incentive salience for these cues, reinforcing future decisions to move (Van Cappellen et al., 2018).

### Permission to Prioritize

Starting from infancy, we learn what to prioritize through socialization within our families, cultures, and larger society. This unconscious process results in deeply rooted beliefs and priorities that influence our daily decisions (Eccles and Wigfield, 2002).

Even after people reframe exercise from a chore to a gift, they may still need to convert it into a priority that justifies making time for it. This can be achieved through perceiving exercise as a type of self-care and permitting themselves to make self-care a priority.

Self-care is often misperceived as selfish and leaves many feeling guilty. For people juggling competing priorities, this can interfere with their exercise motivation. Thus, there is a need to also help individuals rethink exercise as a strategic self-care tactic for supporting their other life priorities.

***Message #3***: “***Give yourself permission to prioritize self-care.***”

The *Give yourself permission to prioritize self-care* message guides people to feel comfortable prioritizing time for physical activity as acts of essential self-care. Similar to the chore-to-gift transformation process, this is also a fundamental resocialization, aimed at helping people reframe exercising as a strategic choice to *fuel what matters most* (Segar, 2015).

#### Empirical support

I learned that even motivated cancer survivors had difficulty prioritizing exercise and self-care back in 1994. Evidence over the subsequent decades establishes the de-prioritization of self-care as a core barrier to exercising, especially among parents and women (Mailey et al., 2014; Segar et al., 2017a; Vrazel et al., 2008). This makes sense because exercising doesn't occur in a vacuum, but within the context of numerous competing daily responsibilities (Gebhardt and Maes, 1998).

Perceiving exercise as compatible with and supporting one's other life priorities is associated with adherence and self-care practices (Zhang et al., 2015). Yet, little is known about how to increase the prioritization of self-care as a motivational gateway to exercising. In addition, my ongoing academic research and experiences coaching clients toward *giving themselves permission to prioritize their exercise as self-care* show that, while possible, this mindset shift can be challenging to achieve due to conflicting societal pressures and competing goals (Segar et al., 2017b).

## Messages are more than just a string of words

Messages are more than just communications. They are a gateway to experiences. They guide people to beliefs, perceptions, experiences, and desires—or lack thereof. Moreover, the need to guide people and populations to perceive and approach exercise in new, more adaptive ways has become a recognized imperative (Segar et al., 2011; Arena et al., 2018; Mailey et al., 2018). “Reframing exercise” was recently adopted as a formal science-based strategy within the United States National Physical Activity Plan to boost population-level physical activity (Physical Activity Alliance, 2023).

The Motivation MAP's three messaging categories, “*Feel Good, Everything Counts*, and *Prioritize Self-care*,” supported by their respective methodologies described in detail elsewhere (Segar, 2015), constitute a comprehensive reframing of exercise (Clifton et al., 2009). They guide individuals to develop new, consciously self-affirming, constraint-reducing, and priority-aligning beliefs that open the door for new experiential possibilities: choosing positive exercise experiences, developing positive affective associations and action tendencies, and noticing and responding to dynamic motivation states.

I designed these three messages to work *together* synergistically because of the specific barriers they aimed to overcome (i.e., 1. exercise is a chore; it feels bad to do and is a “should”, 2. exercise takes too much time and is all-or-nothing, and 3. discomfort with and low competence for prioritizing time for self-care behaviors like exercise; Segar, 2015). Thus, each message may not be impactful if used alone, as other research using single messaging strategies has found (Mailey et al., 2023).

Findings from an earlier NIH-funded evaluation of the long-term impact from the pilot version of The Motivation MAP with its full methodology showed a 65% (*P* < 0.01) increase in physical activity participation from baseline to the study follow-up, which was 9 months or more post-program for 78% of the participants (Segar et al., 2002). It is important to note that this mixed-method evaluation was limited by being a convenience sample with no control group. The results, however, suggested that the intervention did impact the hypothesized mediators of change. *Taking a pleasure-based approach to exercising*, assessed quantitatively, increased from baseline to follow-up by 54% (*P* < 0.01). Qualitative comments suggested that participants transformed physical activity from a chore into an activity that that felt good to do. One participant noted that she started choosing to participate in different activities so she could feel good “*during the process*” of exercising and another said that instead of thinking that she “*had to*” take a walk, she thought about it as something “*to enjoy.*..” Other qualitative comments indicated that they developed more inclusive and flexible definitions of exercise, helping them overcome past absolutist thinking (i.e., “*Everything counts. It all adds up*.”). One participant noted “*Before the class I wouldn't go [to the gym] unless I had a good hour…and after [the intervention] I would go, even if I could only take a 30- or 15-min walk around the track*.” In addition, quantitative data showed a 29% (*P* < 0.01) increase in the *prioritization of self-care* from baseline to follow-up, suggesting that participants may have started internalizing this important value.

This intervention was built for and implemented across real-world community and healthcare settings, giving this past research high external validity. Though The Motivation MAP was developed prior to the emergence of measures (Zenko and Ekkekakis, 2019) and methods (Antoniewicz and Brand, 2016; Maltagliati et al., 2024) that target automatic exercise processes, it aligns with the integration of reflective and experiential processes seen in dual-process theories and constructs within other contemporary behavior change frameworks. For example, the “Move in ways that feel good to you” and “Everything Counts. It all adds up” messages would support the *autonomy* and *competence* human needs posited by self-determination theory. In addition, it maps onto the COM-B model's three core components: capability, opportunity, and motivation (Michie et al., 2011). The “Everything Counts. It all adds up” message supports *capacity* by reducing the perceived and real barriers people have to exercising and it supports *opportunity* by emphasizing how easy it is to claim the opportunities to move that exist all day long. The “Move in ways that feel good to you” message supports *motivation* by giving people permission to autonomously design exercise to deliver positive affective experiences with the intention of helping them develop new positive affective associations (e.g., “tags”) (Brand and Ekkekakis, 2017) that they want to approach. Finally, the “Give yourself permission to prioritize self-care” message may support *capacity* and *motivation* by reducing some of the barriers to prioritizing exercise into daily life as necessary self-care and helping people perceive self-care as a true tactic to fuel themselves for their top priorities.

Because The Motivation MAP was built over 25 years ago for real-world use and was described in detail for non-academic audiences nearly a decade ago (Segar, 2015), many behavioral scientists and interventionists do not know about this theory-based, scientifically supported framework, nor that they can use it within their own research. The Motivation MAP is inherently flexible; each message category and its respective methods can be adapted for different populations and prevention/disease contexts, using formative research with experts and/or community and organizational partners, to identify the terms, concepts, and/or methods that might be most relevant and compelling *to them*. For example, The Motivation MAP's three messaging strategies were adapted for a recent, NIH-funded RO1 intervention targeting cancer prevention, showing promising initial data for engagement (Buis et al., 2024).

Messages matter more than many think; they influence people's exercise frames, beliefs, meanings, experiences, associations, inclinations, and motivation (Hadfield et al., 2023; Segar et al., 2012). The Motivation MAP is a flexible framework to help people reframe and re-experience exercise that is supported by emerging theory, mounting science, and decades of real-world use. It can be adapted to advance and accelerate our next generation of science, practice, and behavioral outcomes.

## References

[B1] Antoniewicz F. Brand R. (2016). Learning to like exercising: evaluative conditioning changes automatic evaluations of exercising and influences subsequent exercising behavior. J. Sport Exerc. Psychol. 2, 138–148. 10.1123/jsep.2015-0125 27385674

[B2] Arena R. McNeil A. Street S. Bond S. Laddu D. R. Lavie C. J.. (2018). Let us talk about moving: reframing the exercise and physical activity discussion. Curr. Probl. Cardiol. 43, 154–179. 10.1016/j.cpcardiol.2017.06.002 29530242

[B3] Berridge K. C. Robinson T. E. Aldridge J. W. (2009). Dissecting components of reward:‘liking',‘wanting', and learning. Curr. Opin. Pharmacol. 9, 65–73. 10.1016/j.coph.2008.12.014 19162544 PMC2756052

[B4] Brand R. Ekkekakis P. (2017). Affective–Reflective Theory of physical inactivity and exercise. Ger. J. Exerc. Sport Res. 48, 48–58 10.1007/s12662-017-0477-9

[B5] Buis L. R. Kadri R. L. Segar M. Poore K. Adwere-Boamah R. Orr J.. (2024). “mHealth to support dietary change for colorectal cancer prevention: early findings on engagement with the MyBestGI app.” in Poster C29, Society of Behavioral Medicine: Annual Meeting (Philadelphia, PA), 13–16.

[B6] Bull F. C. Al-Ansari S. S. Biddle S. Borodulin K. Buman M. P. Cardon G.. (2020). World Health Organization 2020 guidelines on physical activity and sedentary behaviour. *Br. J. Sports Med*. 54, 1451–1462. 10.1136/bjsports-2020-102955 33239350 PMC7719906

[B7] Chan E. T. Li T. E. Schwanen T. Banister D. (2021). People and their walking environments: an exploratory study of meanings, place and times. Int. J. Sust. Transp. 15, 718–729. 10.1080/15568318.2020.1793437

[B8] Chang H. H. Tuan Pham M. (2013). Affect as a decision-making system of the present. J. Cons. Res. 40, 42–63. 10.1086/668644

[B9] Clifton R. Ahmed S. Allen T. Anholt S. Barwise P. Blacket T. (2009). “Brands and branding (the economist series),” in The Economist (New York, NY: Bloomberg Press).

[B10] Conroy D. E. Berry T. R. (2017). Automatic affective evaluations of physical activity. Exerc. Sport Sci. Rev. 45, 230–237. 10.1249/JES.0000000000000120 28704217

[B11] Cooke-Jackson A. Rubinsky V. (2023). Extending the roots of memorable messages: A comprehensive review and forecast of memorable message literature and theory. Health Commun. 38, 2676–2686. 10.1080/10410236.2022.2105620 35898109

[B12] Eccles J. S. Wigfield A. (2002). Motivational beliefs, values, and goals. Annu. Rev. Psychol. 53, 109–132. 10.1146/annurev.psych.53.100901.135153 11752481

[B13] Gebhardt W. A. Maes S. (1998). Competing personal goals and exercise behaviour. Percept. Mot. Skills 86, 755–759. 10.2466/pms.1998.86.3.755 9656267

[B14] Gray J. B. Harrington N. G. (2011). Narrative and framing: A test of an integrated message strategy in the exercise context. J. Health Commun. 16, 264–281. 10.1080/10810730.2010.529490 21058142

[B15] Hadfield J. I. Guerra-Reyes L. Huber L. Major L. Kennedy-Armbruster C. (2023). The message matters: Advertisement framing and college women's beliefs toward exercise. J. Am. College Health 1–12. 10.1080/07448481.2022.2155457 36595623

[B16] Knox E. C. Webb O. J. Esliger D. W. Biddle S. J. Sherar L. B. (2014). Using threshold messages to promote physical activity: implications for public perceptions of health effects. Eur. J. Public Health 24, 195–199. 10.1093/eurpub/ckt060 23729481

[B17] Kwan B. M. Stevens C. J. Bryan A. D. (2017). What to expect when you're exercising: An experimental test of the anticipated affect–exercise relationship. Health Psychol. 36:309. 10.1037/hea0000453 27991804 PMC6317525

[B18] Mailey E. L. Dlugonski D. Besenyi G. M. Gasper R. Slone S. (2023). Effects of a single message exposure on exercise motivation and behavior among adults aged 30-45. Int. J. Health Prom. Educ. 61, 83–97. 10.1080/14635240.2022.2031250

[B19] Mailey E. L. Dlugonski D. Hsu W. W. Segar M. (2018). Goals matter: Exercising for well-being but not health or appearance predicts future exercise among parents. J. Phys. Activity Health 15, 857–865. 10.1123/jpah.2017-0469 30314419

[B20] Mailey E. L. Hsu W. W. (2019). Is a general or specific exercise recommendation more effective for promoting physical activity among postpartum mothers? J. Health Psychol. 24, 964–978. 10.1177/1359105316687627 28810385

[B21] Mailey E. L. Huberty J. Dinkel D. McAuley E. (2014). Physical activity barriers and facilitators among working mothers and fathers. BMC Public Health 14, 1–9. 10.1186/1471-2458-14-657 24974148 PMC4227023

[B22] Maltagliati S. Sarrazin P. Muller D. Fessler L. Ferry T. Wiers R. W.. (2024). Improving physical activity using a single personalized consequence-based approach-avoidance training: effects on self-reported behaviors, attitudes, and choices. Psychol. Sport Exerc. 70, 1–10. 10.1016/j.psychsport.2023.102565 37979927

[B23] Michie S. van Stralen M. M. West R. (2011). The behaviour change wheel: a new method for characterising and designing behaviour change interventions. Implement. Sci. 6, 1–11. 10.1186/1748-5908-6-42 21513547 PMC3096582

[B24] Physical Activity Alliance (2023). Media and Communications. Available at: https://paamovewithus.org/media-and-communications/ (accessed May 29, 2024).

[B25] Segar M. L. (2015). No Sweat: How the Simple Science of Motivation Can Bring you a Lifetime of Fitness. New York: Amacom.

[B26] Segar M. L. (2022). The Joy Choice: How to Finally Achieve Lasting Changes in Eating and Exercise, New York: Hachette Go.

[B27] Segar M. L. Eccles J. S. Peck S. C. Richardson C. R. (2007). Midlife women's physical activity goals: sociocultural influences and effects on behavioral regulation. Sex Roles 57, 837–849. 10.1007/s11199-007-9322-1

[B28] Segar M. L. Eccles J. S. Richardson C. R. (2011). Rebranding exercise: closing the gap between values and behavior. Int. J. Behav. Nutr. Phys. Activity 8, 1–14. 10.1186/1479-5868-8-94 21884579 PMC3180298

[B29] Segar M. L. Guérin E. Phillips E. Fortier M. (2016). From a vital sign to vitality: selling exercise so patients want to buy it. Curr. Sports Med. Rep. 15, 276–281. 10.1249/JSR.0000000000000284 27399825

[B30] Segar M. L. Heinrich K. M. Zieff S. G. Lyn R. Gustat J. Perry C. K.. (2017a). What walking means to moms: Insights from a national sample to frame walking in compelling ways to low-income urban mothers. J. Transp. Health 5, 5–15. 10.1016/j.jth.2016.06.004

[B31] Segar M. L. Jayaratne T. Hanlon J. Richardson C. R. (2002). Fitting fitness into women's lives: effects of a gender-tailored physical activity intervention. Women's Health Issues 12, 338–347. 10.1016/S1049-3867(02)00156-1 12457574 PMC2578875

[B32] Segar M. L. Katch V. L. Roth R. S. Garcia A. W. Portner T. I. Glickman S. G.. (1998). The effect of aerobic exercise on self-esteem and depressive and anxiety symptoms among breast cancer survivors. Oncol. Nurs. Forum 25, 107–113. 9460778

[B33] Segar M. L. Marques M. M. Palmeira A. L. Okely A. D. (2020). Everything counts in sending the right message: science-based messaging implications from the 2020 WHO guidelines on physical activity and sedentary behaviour. Int. J. Behav. Nutr. Phys. Activity 17:135. 10.1186/s12966-020-01048-w 33148305 PMC7643438

[B34] Segar M. L. Taber J. M. Patrick H. Thai C. L. Oh A. (2017b). Rethinking physical activity communication: using focus groups to understand women's goals, values, and beliefs to improve public health. BMC Public Health 17, 1–13. 10.1186/s12889-017-4361-1 28521756 PMC5437577

[B35] Segar M. L. Updegraff J. A. Zikmund-Fisher B. J. Richardson C. R. (2012). Physical activity advertisements that feature daily well-being improve autonomy and body image in overweight women but not men. J. Obes. 2012:354721. 10.1155/2012/354721 22701782 PMC3373161

[B36] Smith S. W. Hamel L. M. Kotowski M. R. Nazione S. Laplante C. Atkin C. K.. (2010). Action tendency emotions evoked by memorable breast cancer messages and their association with prevention and detection behaviors. *Health Commun*. 25, 737–746. 10.1080/10410236.2010.521916 21153990 PMC3059500

[B37] Spruijt-Metz D. (1995). Personal incentives as determinants of adolescent health behavior: the meaning of behavior. Health Educ. Res. 10, 355–364. 10.1093/her/10.3.355 10158028

[B38] Stevens C. J. Baldwin A. S. Bryan A. D. Conner M. Rhodes R. E. Williams D. M. (2020). Affective determinants of physical activity: a conceptual framework and narrative review. Front. Psychol. 11:568331. 10.3389/fpsyg.2020.568331 33335497 PMC7735992

[B39] Stults-Kolehmainen M. A. Gilson T. A. SantaBarbara N. McKee P. C. Sinha R. Bartholomew J. B.. (2023). Qualitative and quantitative evidence of motivation states for physical activity, exercise and being sedentary from university student focus groups. Front. Sports Active Living 5:1033619. 10.3389/fspor.2023.1033619 37025458 PMC10071436

[B40] The Framing Institute (2005). News Framing: Theory and Typology. Available at: https://www.frameworksinstitute.org/tools-and-resources/framing-101/ (accessed May 29, 2024).

[B41] Van Cappellen P. Rice E. L. Catalino L. I. Fredrickson B. L. (2018). Positive affective processes underlie positive health behaviour change. Psychol. Health 33, 77–97. 10.1080/08870446.2017.1320798 28498722 PMC5682236

[B42] Vrazel J. Saunders R. P. Wilcox S. (2008). An overview and proposed framework of social-environmental influences on the physical-activity behavior of women. Am. J. Health Promot. 23, 2–12. 10.4278/ajhp.06070999 18785368

[B43] Walters A. J. Tomasone J. R. Latimer-Cheung A. E. (2022). An experimental test of a generic messaging approach for the Canadian 24-hour movement guidelines for adults. J. Health Commun. 27, 8–16. 10.1080/10810730.2021.2025175 35109769

[B44] Warburton D. E. Bredin S. S. (2016). Reflections on physical activity and health: what should we recommend? Can. J. Cardiol. 32, 495–504. 10.1016/j.cjca.2016.01.024 26995692

[B45] Zahrt O. H. Crum A. J. (2020). Effects of physical activity recommendations on mindset, behavior and perceived health. Prevent. Med. Rep. 17:101027. 10.1016/j.pmedr.2019.101027 31921575 PMC6948259

[B46] Zenko Z. Ekkekakis P. (2019). Internal consistency and validity of measures of automatic exercise association. Psychol. Sport Exerc. 43, 4–15. 10.1016/j.psychsport.2018.12.00531469367

[B47] Zhang K. M. Dindoff K. Arnold J. M. O. Lane J. Swartzman L. C. (2015). What matters to patients with heart failure? The influence of non-health-related goals on patient adherence to self-care management. Patient Educ. Counsel. 98, 927–934. 10.1016/j.pec.2015.04.011 25979423

